# About time? The role of time perspective in the priority for positive over negative emotion in attention

**DOI:** 10.1080/02699931.2026.2675414

**Published:** 2026-06-02

**Authors:** Briana L. Kennedy, Mara Mather

**Affiliations:** a School of Psychological Science, University of Western Australia, Perth, WA, Australia; b Leonard Davis School of Gerontology, University of Southern California, Los Angeles, CA, USA; c Department of Psychology, University of Southern California, Los Angeles, CA, USA; d Department of Biomedical Engineering, University of Southern California, Los Angeles, CA, USA

**Keywords:** Positivity effect, attention, emotion, aging

## Abstract

Compared to younger adults, older adults often prioritise processing positive information and deprioritize processing negative information. Known as the positivity effect, its mechanisms remain debated. One explanation of the positivity effect is that older adults are influenced by a sense of limited time left in life to focus more on emotional well-being compared with younger adults. Previous research shows that experimentally manipulating time perspective can mimic the positivity effect in younger adults, however, such findings have relied on designs where participants can reflect on emotional images they are viewing. In the current study, we examined if time perspective manipulations would similarly elicit positivity effect patterns in younger adults using an emotion-attention task with a rapid stimuli display. Across three experiments (*N* = 236, *N* = 431, and *N* = 259), we found that time perspective had little influence on attentional biases toward positive over negative emotional stimuli in younger adults. Additionally, when we incorporated a memory task into the design, effects of time perspective were not consistent with previous findings – in fact, patterns were in the opposite direction. We discuss these results as they relate to theories and the advantages and disadvantages of tasks used to examine the positivity effect.

Older adults have a propensity to prioritise positive information and deprioritize negative information compared to younger adults (Charles et al., 2003; Mather & Carstensen, 2005; Reed et al., 2014; Reed & Carstensen, 2012). This pattern, known as the positivity effect, is observed over a spectrum of cognitive processing stages, including attention, memory, and decision making (Carstensen & DeLiema, 2018; Carstensen & Reynolds, 2023; Charles et al., 2003; Isaacowitz & Blanchard-Fields, 2012; Löckenhoff & Carstensen, 2007; Mather et al., 2005; Niu et al., 2024; Reed & Carstensen, 2012). For example, compared to younger adults, older adults tend to remember positive images better and negative images worse (Charles et al., 2003; Mather & Knight, 2005). The mechanisms that drive the positivity effect remain under debate (Barber et al., 2020; Barber & Kim, 2022; Charles, 2010; Ferguson & Leal, 2022; Gronchi et al., 2018; Isaacowitz, 2022; Kapoor et al., 2025; Kryla-Lighthall & Mather, 2009; Labouvie-Vief et al., 2010; Mather, 2024; Petro et al., 2021; Reed & Carstensen, 2012; Sakaki et al., 2019). Candidate theories often diverge in whether the positivity effect requires strategic or automatic processing (Barber et al., 2020; Mather, 2024; Mather & Knight, 2005), which makes tasks that limit strategic processing especially well placed to understand how the positivity effect operates.

The most influential theory regarding the mechanisms of the positivity effect has been the “socioemotional selectivity theory”, which proposes that age differences in time perspective are responsible for the positivity effect (Carstensen & Reynolds, 2023; Mather & Carstensen, 2005; Reed & Carstensen, 2012). By this account, older adults tend to perceive a limited time horizon left in life, and therefore prioritise positive and emotionally meaningful information rather than negative information no longer needed for future learning (e.g. Carstensen, 2006). Other theories consider processing that is more automatic, such as the dynamic integration theory (Labouvie-Vief et al., 2010), which instead proposes that the positivity effect occurs because older adults automatically inhibit processing negative information because it is harder or more complicated to process. A recent proposal argues that the age-related positivity effect could result from a side effect of the aging brain attempting to down-regulate hyperactive noradrenergic and sympathetic activity (Mather, 2024). Still other accounts propose multiple mechanisms, with separable automatic and strategic influences involved in the positivity effect (e.g. Barber et al., 2020; Gronchi et al., 2018).

Understanding the mechanisms in the positivity effect has implications beyond understanding age-related change. Being able to induce a bias toward positive information and away from negative information could be helpful for individuals who struggle with negative emotion (e.g. Bar-Haim, 2010; Hakamata et al., 2012; MacLeod et al., 2002; Macleod & Mathews, 2012). Young adults in particular tend to have a negativity bias – prioritising negative stimuli over neutral or positive stimuli – compared to the positivity bias in older adults (Baumeister et al., 2001; Carstensen & DeLiema, 2018). This makes producing a positivity bias in younger adults both an important avenue to test potential underlying mechanisms of the positivity effect, as well as a potential way to develop strategies to help others view the world with a more positive lens.

Young adults can demonstrate the positivity effect when they have limited time horizons. For example, people who are imminently moving to another location (Fredrickson & Carstensen, 1990), or are patients with critical illnesses (Carstensen & Fredrickson, 1998), have shown positivity effects similar to older adults (see also Carstensen & Reynolds, 2023). Similarly, students at the end of their university experience compared to those at the beginning have shown patterns similar to the positivity effect (Pruzan & Isaacowitz, 2006). Such findings are consistent with the idea that the positivity effect results from a changed time horizon perspective (Carstensen, 2006). Further evidence comes from work that experimentally manipulates time perspective. Previous research demonstrates that inducing a more “limited” time perspective in younger adults can make them mimic the older adult positivity effect bias (Barber et al., 2016; Kellough & Knight, 2012; Shavit et al., 2023; Zsoldos & Hot, 2024). For example, when younger adults were given prompts to consider their time left in life as more limited, they remembered relatively more positive images and fewer negative images compared with participants that were given prompts to consider time left in life as more expansive (Barber et al., 2016).

If time horizons are responsible for positivity effects, a task that does not require strategic processing could reveal whether deliberate processing mechanisms are necessary for the effects to occur. However, previous experiments that used time perspective manipulations have also tended to use tasks that allow time for higher-level processing in the measurement of the effect. For example, even when time perspective is explored in attentional tasks, such tasks often rely on lengthy time intervals (Pruzan & Isaacowitz, 2006; Zsoldos & Hot, 2024) that can allow time-dependent, higher-level processing. For example, Zsoldos and Hot (2024) recently conducted a time manipulation experiment using a dot probe task. Using reaction time as a measure of how much attention biased to emotional images, they found that when a probe appeared 500 ms after the images, younger adults with a limited time perspective and older adults showed a bias toward positive and away from negative images, but younger adults in a control condition did not. Such findings indicate that the time perspective manipulation can influence attention, although 500 ms still allows for substantial processing to occur between images and the probe.

One task well-positioned to examine emotion effects in attention is the emotion-induced blindness task (Kennedy & Most, 2012; Most et al., 2005). In this task, participants tend to perform worse when identifying a target image that appears soon after an emotionally powerful image, compared to an emotionally neutral image in a rapid stream of images (Most et al., 2005). Emotion-induced blindness patterns also reveal the way emotional stimuli interrupt awareness across time: emotionally powerful stimuli tend to impair awareness for a subsequent target image when the target appears at an early lag (e.g. 200 ms after the emotional distractor) but less so when the target appears at a later lag (e.g. 400 ms after the emotional distractor; e.g. Kennedy & Most, 2015). Across three experiments, a recent study revealed age differences in emotion-induced blindness patterns consistent with a positivity effect, such that older adults were more distracted by positive distractors and less distracted by negative distractors compared to younger adults at early lags (Kennedy et al., 2020). We subsequently replicated the age-by-valence interaction in emotion-induced blindness patterns in two other studies (Fox et al., 2026; Kennedy & Mather, 2024). In the current study, we examined whether time perspective manipulations in younger adults can shift positivity biases in attention during the emotion-induced blindness task. Examining biases in this particular task is of particular interest since it has been demonstrated to elicit an age-related positivity effect (Fox et al., 2026; Kennedy et al., 2020; Kennedy & Mather, 2024), yet it limits time for influences of higher-level processing after stimulus presentation (Kennedy & Most, 2012).

Using a time perspective manipulation task and an emotion-induced blindness task, we predicted that a limited time horizon perspective would make young adult participants perform worse at identifying the target on trials with positive distractors compared to negative distractors compared to participants in the expansive or control conditions.

## Experiment 1

### Method

#### Participants

Two hundred forty-eight younger adults completed Experiment 1 (169 females, 79 males, with ages ranging from 18 to 32; *M* = 20.0 years old). All participants were undergraduate students from the University of Southern California with an average of 13.9 years of education (*SD* = 1.3 years) and were recruited online through the university’s SONA research participation system between February 2018–May 2018. Eligibility requirements included that participants were healthy adults 18 years old or over, did not have any serious chronic illness, did not have cognitive impairment, reported normal or corrected vision, spoke English fluently, and were not currently taking beta-blocker medication. Our sample size was selected to detect a small to medium effect in our predicted within-between interactions. Our target *N* provided power to observe effect sizes of *f* = 0.10, with *α* = .05 and power (1 – β) = 0.8 (calculated with G*Power; Faul et al., 2007). After data collection, we excluded data from 12 participants due to poor performance or failing to complete the time perspective writing task (see Data Screening); the final sample was comprised of 236 participants. The experiment was approved by the University of Southern California Institutional Review Board, protocol UP-12–00019. Participants received course credit for completion of the experiment, which took approximately 30 min to complete.

#### Materials

The experiment was programmed with Inquisit (Inquisit 5, 2016) and run online. Participants signed up for the study via a link that allowed them to complete the experiment exactly once to earn course credit and completed the experiment on their own computers in their own time and in their own space. The experimental programme and anonymized data are available on OSF: https://osf.io/da84u. Experiment 1 was not preregistered.

##### Time perspective writing task.

We used a time perspective writing task that was the same as that developed by Barber and colleagues (2016), and that is available in their manuscript. Participants were randomly assigned to one of three time-perspective conditions: expansive, limited, or control. Each participant was asked to answer questions based on a prompt about how to imagine their future. In the expansive condition, participants were tasked to consider how people keep living longer and answer questions as though they were planning to live until 120 years old. In the limited condition, participants were tasked to consider that people can never know when life will end and answer questions as though they were planning to live for only six more months in good health. Finally, in the control condition, participants answered questions unrelated to their future. Participants in all three conditions were asked to answer four open-ended questions phrased in a way that matched the prompt, related to things such as how they would spend money and their goals. Participants were told that answering all four questions should take about five minutes (~1 min per question).

##### Emotion-induced blindness task.

**Figure 1.**
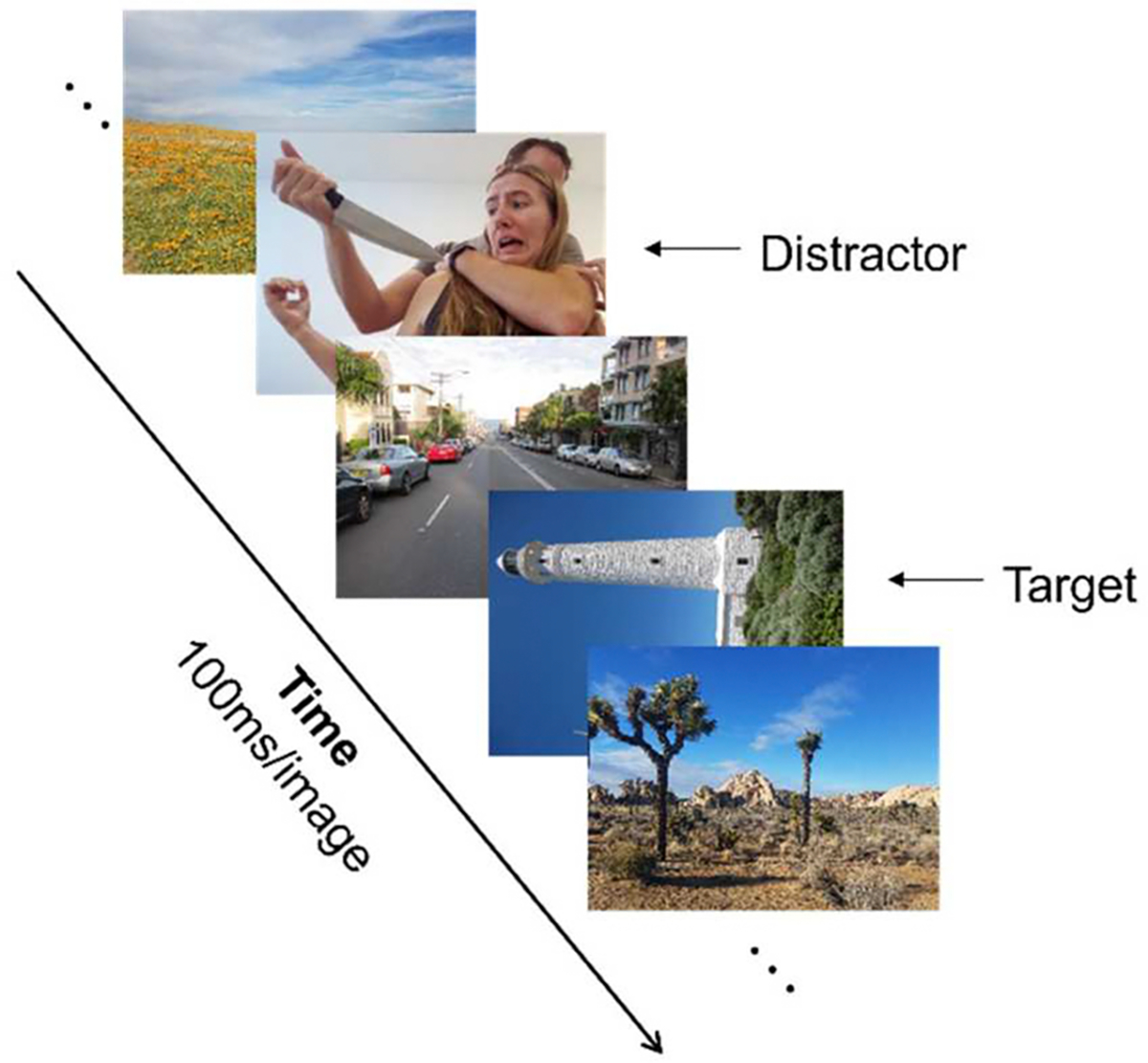
Schematic of the emotion-induced blindness trial design. Notes: This figure depicts part of an example negative, lag 2 trial. Images used in this figure are for example only and were not those used in the formal experiment.

An emotion-induced blindness task (adapted from Most et al., 2005) contained 192 experimental trials split between 4 blocks of 48 trials. On every trial, images were presented in a rapid serial visual presentation (RSVP; see Figure 1). In the RSVP, images were shown one at a time, displayed at a rate of 100 ms per image, and immediately replaced the previous image. Each trial contained 17 images in total, composed of one distractor, one target image, and 15 filler images. All images were coloured, 320-pixel × 240-pixel photographs that were displayed in the centre of the monitor against a white background. Images were processed using the SHINE Toolbox (Willenbockel et al., 2010) to minimise differences in luminance between images.

There were four distractor categories in the experiment: emotionally negative, emotionally neutral, emotionally positive, and no distractor. We selected 36 images per category from the International Affective Picture System (IAPS) based on normative ratings of valence (negative = 1 to positive = 9) and arousal (low arousal = 1 to high arousal = 9). Emotionally negative, neutral, and positive images were the same as those used by Kennedy et al. (2020), Experiment 2, and the list of images used is available in our additional material on OSF: https://osf.io/da84u.

Target and filler images were chosen from previous emotion-induced blindness studies and depicted landscape scenes (Kennedy et al., 2020; Most et al., 2005). Target images were rotated 90 degrees to the left and 90 degrees to the right, and the experiment included 64 images per rotated direction. There were an additional 252 images that served as filler images in the RSVP stream, all of which appeared in an upright orientation.

On any trial, the distractor could appear in serial positions 3, 4, 5, or 6. Targets always appeared after the distractor – on half of the trials, the target was the second image after the distractor (lag 2) and on the other half of the trials, the target was the fourth image after the distractor (lag 4). Lag 2 performance is typically considered a lag to observe the greatest impact from emotional distractors in the emotion-induced blindness task, whereas lag 4 is typically about when performance recovers from emotion-induced impairment (Kennedy & Most, 2015).

Participants were told that their task was to watch the rapid stream of images on every trial and determine the direction of one rotated picture in the stream of otherwise upright images. They were told that sometimes they would see pictures of people or animals, but that they should ignore them, as these would never be the target. After the RSVP stream, participants saw a prompt that read, “Rotated left (F) or right (J)?” This screen remained until participants made their response by indicating it via a corresponding keypress. They saw their performance accuracy on each trial via a message that said “Correct!” or “Incorrect,” which displayed immediately after their response for one second. The programme progressed to a fixation cross presented for one second, and then the next trial started automatically. The emotion-induced blindness task took about 15 min to complete.

A practice session oriented participants to the task design. There were eight practice trials that started at a rate of 200 ms per image and slowly increased to the experimental speed of 100 ms per image. No distractors appeared in the practice trials.

#### Questionnaires

##### Mood.

Mood was measured via a sliding scale. Participants were asked to indicate their current mood by responding to the prompt, “Please indicate your current mood on a scale from 0 (very negative mood) to 100 (very positive mood)”. Participants made their response with their mouse using a slider, and values on the scale were shown to participants, which ranged from 0 to 100. The mood measurement was taken twice in the experiment to determine the impact from the writing task manipulation on mood.

##### Life progress.

To measure life progress, participants were asked to move a slider on a scale, with the instructions, “Please move the slider to how far you have progressed in your life, where the left-hand side of the scale represents the beginning of your life”. The slider scale had anchors of “Beginning” and “End” and values on the scale were hidden from participants but ranged from 0 to 100.

##### Future Time Perspective.

On the future time perspective scale (Carstensen & Lang, 1996), participants were asked to rate their perception of their future across three statements on a scale from 1 (strongly disagree) to 7 (strongly agree). The statements included “My future seems infinite to me”, “I could do anything I want in my future”, and “Most of my life lies ahead of me”. A higher score on this questionnaire represented a more expansive view of one’s future.

##### Demographics.

Participants were asked to provide their sex, age, year they were born, ethnicity, race, and years of education.

Overall, the questionnaires took about 5 min to complete.

##### Procedure.

Participants signed up for the experiment through the university Sona system and accessed the Inquisit study via a URL link. After reading the consent form and instructions, participants completed practice emotion-induced blindness trials to acquaint themselves with the task. Participants then completed their baseline mood measurement before engaging in the writing task. Immediately after the writing task, participants indicated their mood again (post-writing task mood), and then completed the emotion-induced blindness task. After completing the emotion-induced blindness task, participants completed the life progress rating, the future time perspective questionnaire, and the demographics questionnaire. At the end of the study, participants were debriefed about the goals of the research.

### Results

#### Data screening

Data from eleven participants were excluded for overall performance accuracy < 55% (a standard cutoff we use; see Kennedy et al., 2020). Data from one additional participant was removed because the participant did not complete the time perspective writing task or indicate their mood. Our final sample thus included 236 participants, with 82 participants in the “expansive” condition, 78 in the “limited” condition, and 76 in the “control” condition.

We analysed all data using null hypothesis significance testing; however, because several comparisons of interest yielded null effects – especially interactions indicative of the positivity effect – we conducted complementary Bayesian analyses to further quantify the strength of evidence for or against key effects. We did this by replicating ANOVAs and isolating Bayes factors for interaction terms by comparing models with and without the relevant interactions. Bayes factors were computed with the BayesFactor package in R (Morey & Rouder, 2023), which estimate marginal likelihoods via Monte Carlo sampling. Participant numbers were included as random factors to account for repeated measures. Bayes factors were interpreted based on guidelines provided in Wetzels et al. (2011).

#### Writing task attention check

We examined responses on the writing task to ensure that participants engaged with the writing task during the experiment. All participants in the final sample responded with task-appropriate responses to the prompts, confirming that they read the prompts and answered accordingly. On average, participants took 155.9 s to complete the writing task and wrote an average of 336.7 characters to answer all four prompts, however, the time and character length varied greatly between participants (time: *SD* = 142.9 s; range: 25.2–1189 s; characters: *SD =* 274.5 characters, range: 26–1939 characters). These differences were driven largely by the control condition: participants spent significantly less time and wrote significantly fewer characters in the control condition (time: *M* = 79.7 s, *SD* = 49.6 s; characters: *M* = 142.9 characters, *SD* = 100.4 characters) compared to both the expansive (time: *M =* 174.6 s, *SD* = 120.3 s; characters: *M* = 425.8 characters, *SD* = 302.9 characters) and limited (time: *M* = 210.5 s, *SD* = 188.6 s; characters: *M* = 425.8 characters, *SD* = 262.4 characters) conditions, *ps* < .001. Notably, there were no significant differences in time spent or character count between the expansive and limited conditions, *ps* ≥ .265, confirming that participants engaged similarly on the two time-manipulation conditions.

#### Effect of time manipulation on emotion-induced blindness

**Figure 2.**
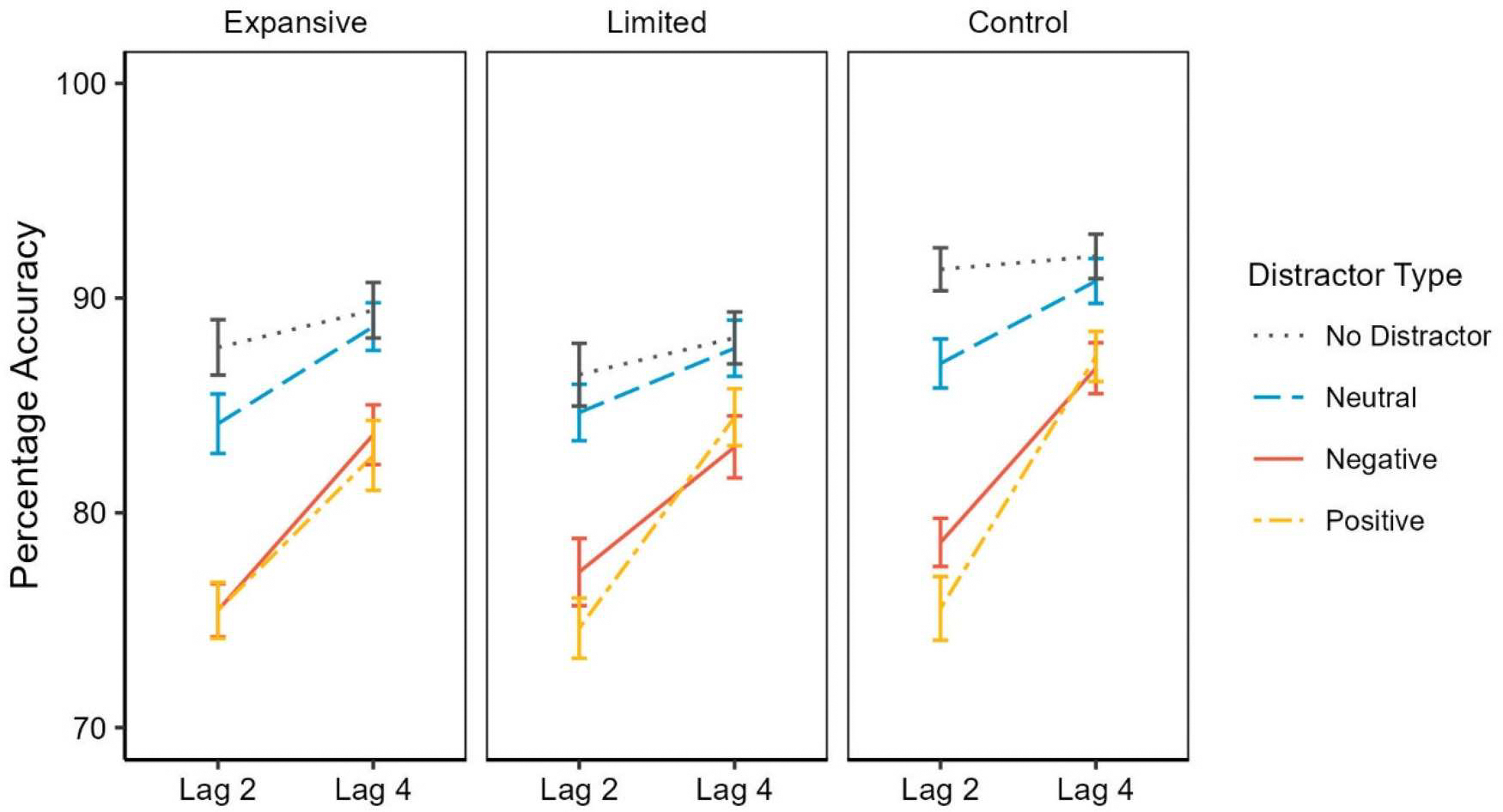
Experiment 1 emotion-induced blindness patterns for each time manipulation group. Notes: Error bars represent between-subject standard error. For reference, means for each condition are also reported in the Supplemental Material Table S1.

A 4 (distractor type: positive, negative, neutral, no distractor) × 2 (lag: 2, 4) × 3 (time manipulation condition: expansive, limited, control) mixed ANOVA revealed a significant main effect of distractor type, *F*(3,699) = 155.70, *p* < .001, $\eta_{p}^{2}=.40$, with worse performance overall on positive and negative trials compared to neutral and no distractor trials (see Figure 2). There was also a significant main effect of lag, *F*(1,233) = 217.77, *p* < .001, $\eta_{p}^{2}=.48$, and distractor type × lag interaction, *F*(3,699) = 31.71, *p* < .001, $\eta_{p}^{2}=.12$, such that performance was worse overall on lag 2 compared to lag 4 trials, and performance after positive and negative distractors recovered between lag 2 and lag 4 more so than neutral and no distractor trials. Such patterns are consistent with the emotion-induced blindness effect. However, when it came to the impact of time manipulation condition, there was no main effect, *F*(2,233) = 2.47, *p* = .087, $\eta_{p}^{2}=.02$, nor was there a significant distractor type × time manipulation condition, lag × time manipulation condition, or three-way distractor type × lag × time manipulation condition (*F*s ≤ 1.83, *ps* ≥ .091, $\eta_{p}^{2}s\leq .02$). Thus, the time manipulation condition did not have the predicted impact on performance – performance was similar across time manipulation conditions.

#### Post-hoc analysis: expansive and limited emotional trials only

Our null results indicated that time manipulation did not make an impact on performance in emotion-induced blindness. However, we conducted exploratory post-hoc analyses to determine whether the time perspective manipulation may have had an impact on the conditions typically used for positivity effect comparisons. After all, the positivity effect is operationally defined as the specific comparison of negative and positive conditions and between younger adults (expansive) and older (limited) adults. By isolating the comparisons to only those conditions in a post-hoc exploratory analysis, we could more closely match experiments that typically observe a positivity effect.

When we restricted our analysis to a 2 (distractor type: positive, negative) × 2 (lag: 2, 4) × 2 (time manipulation condition: expansive, limited) mixed ANOVA, and made no corrections for multiple comparisons, there was still no significant distractor type × lag × time manipulation condition, *F*(1,158) = 3.80, *p* = .053, $\eta_{p}^{2}=.02$. A subsequent Bayesian ANOVA with the same distractor type × lag × time manipulation factors revealed weak support for the null for the three-way interaction when it was isolated in the model, BF_10_ = 0.77. Thus, the Bayesian analysis provided further support (albeit weak) that there was no significant difference in the effect from negative and positive distractors between the expansive and the limited conditions.

#### Questionnaire results

We next examined questionnaire data from participants by time manipulation condition, depicted in Table 1.

There was no difference between time manipulation conditions (expansive, limited, control) across the variables of baseline mood, post-writing task mood, life progress, or future time perspective (*ps* ≥ .143). There was, however, a significant effect of time manipulation condition × mood change, *F*(2,235) = 5.27, *p* = .006, *η*^*2*^ = .04. Participants in the limited condition reported a decrease in mood compared to the expansive condition after completing the writing task, *t*(158) = 2.97, *p* = .003, *d* = .47. There was no difference in change of mood between the expansive condition and control condition, *t*(156) = 1.56, *p* = .120, *d* = .25, nor the limited condition and control condition, *t*(152) = 1.79, *p* = .076, *d* = .29. Thus, mood seemed negatively affected by the limited condition (in a way similar to patterns found by Barber et al., 2016), but no other questionnaire measures were affected by the writing task manipulation. In secondary exploratory analyses, we examined how individual differences from questionnaire responses related to performance, as well as how the time of semester that participants completed the study related to their performance. We report these exploratory analyses in the Supplemental Material for interested readers.

### Discussion

In Experiment 1, time perspective manipulations did not significantly impact emotion-induced blindness patterns.

## Experiment 2

In Experiment 2, our main aim was to replicate the design of Experiment 1 with a greater sample size. We wanted to examine if doing so would reveal if younger adults’ time perspective might make them more distracted by positive stimuli compared to negative stimuli. In Experiment 2, we further examined the role of individual differences. Time and affect, in particular, are constructs at the heart of the time perspective predictions, so we examined whether these individual differences predicted performance on the task.

### Method

#### Participants

Four hundred fifty-one participants took part in Experiment 2. Data from five participants were excluded because they did not complete the full experiment. Participants were undergraduate students at the University of Southern California recruited on the university Sona system between September 13, 2018–January 31, 2019. The sample was comprised of 289 females, 156 males, and one participant who preferred not to respond about their sex, with an age range of 18–34 (*M* = 20.0, *SD* = 1.8) and an average of 14.1 years of education (*SD* = 1.3 years). The design and analysis plan for Experiment 2 was preregistered at https://osf.io/pbrz7.^1^

We aimed to collect a sample size of 498 participants – 166 in each of the three time perspective conditions. This accommodated a sample size of 156 participants per time condition group, which was determined using data from our first experiment (80% power, *α* = .05, computed with G*Power; Faul et al., 2007), plus an additional 10 participants per group to accommodate for participants that did not complete the full task and to allow for unevenness in group assignments in the online study. However, we were selectively recruiting participants across certain times of the semester, so were limited by the number of participants that completed the study in the times allocated. After data collection, we screened out 15 participants for poor performance (see Data Screening), and in total, our final sample comprised 431 participants. Experiment 2 took about 45 min to complete and was approved by the University of Southern California Institutional Review Board, protocol UP-12–00019. Participants were awarded course credit for their participation.

#### Materials

The experimental programme and anonymized data for Experiment 2 are available on OSF: https://osf.io/da84u. The same time perspective writing task, emotion-induced blindness task, and questionnaires were used in Experiment 2 as they were in Experiment 1; however, we also introduced five additional questionnaires to capture individual differences of interest. These included the Positive and Negative Affect Schedule (PANAS; Watson & Clark, 1994), the Big Five Inventory (BFI; John et al., 1991, 2008), the Revised Life Orientation Test (LOT-R; Scheier et al., 1994), a subset of questions from the Behaviourally Anchored Response Scales of Time Urgency (as a measure of time orientation; Landy et al., 1991), and a novel set of seven questions related to time organization (available in our additional materials available on OSF: https://osf.io/da84u). The additional questionnaires took about 15 min to complete. The results from these individual difference questionnaires fell outside of the scope of our main research aims, along with analyses related to semester effects, but are available in our Supplementary Material.

#### Procedure

The procedure was the same as Experiment 1, except for additional questionnaires that participants completed at the end of the experiment. After the emotion-induced blindness task, participants completed the series of questionnaires in the order of (1) future perspective, (2) PANAS, (3) BFI, (4) LOT-R, (5) time orientation, (6) time organization, (7) demographics.

### Results

#### Data screening

Data from fifteen participants were excluded for overall performance accuracy < 55%, making the final sample 431 participants. There were 144 participants in the “expansive” condition, 148 in the “limited” condition, and 139 in the “control” condition.

#### Writing task attention check

All participants responded to the time manipulation writing task with task-appropriate responses. Participants took an average of 158.7 s and an average of 327.1 characters to answer all four prompts in the writing task, with a large spread between participants on both measures (time: *SD* = 224.6 s; range: 22.7–3799.6 s; characters: *SD =* 256.5 characters, range: 17–1550 characters). Like Experiment 1, participants in the control condition spent significantly less time and wrote significantly fewer characters (time: *M* = 89.8 s, *SD* = 119.8 s; characters: *M* = 136.7 characters, *SD* = 84.5 characters) compared to both the expansive (time: *M =* 175.1 s, *SD* = 146.6 s; characters: *M* = 395.7 characters, *SD* = 246.6 characters) and limited (time: *M* = 207.5 s, *SD* = 325.5 s; characters: *M* = 439.1 characters, *SD* = 274.0 characters) conditions, *ps* ≤ .004. There were also no significant differences in time spent or character count between the expansive and limited conditions, *ps* ≥ .278, which suggests participants engaged in the task similarly across the two time manipulation conditions.

#### Effect of time manipulation on emotion-induced blindness

**Figure 3.**
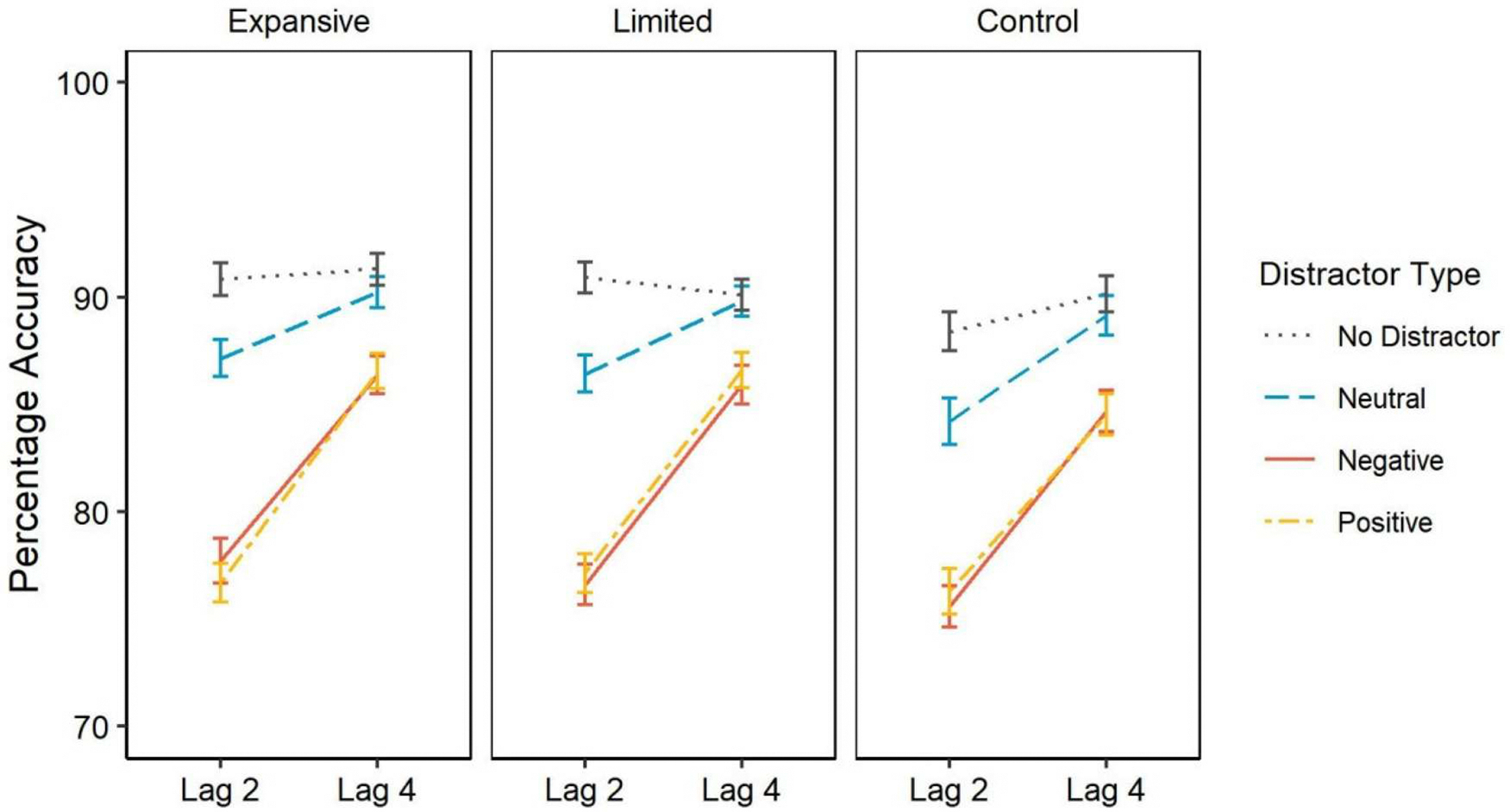
Experiment 2 emotion-induced blindness results for each time manipulation group. Notes: Error bars represent between-subject standard error. For reference, means for each condition are also reported in the Supplemental Material Table S2.

A 4 (distractor type: positive, negative, neutral, no distractor) × 2 (lag: 2, 4) × 3 (time manipulation condition: expansive, limited, control) mixed ANOVA revealed a significant main effect of distractor type, *F*(3,1284) = 312.75, *p* < .001, $\eta_{p}^{2}=.42$, main effect of lag, *F*(1,428) = 529.34, *p* < .001, $\eta_{p}^{2}=.55$, and distractor type by lag interaction, *F*(3,1284) = 79.53, *p* < .001, $\eta_{p}^{2}=.16$, each confirming that there was overall an emotion-induced blindness effect (see Figure 3). However, there was no main effect of time manipulation condition, distractor type × time manipulation condition interaction, lag × time manipulation condition interaction, or distractor type × lag × time manipulation condition interaction (*F*s ≤ 1.79, *ps* ≥ .168, $\eta_{p}^{2}s\leq .008$). In other words, like Experiment 1, time manipulation conditions had no significant impact on emotion-induced blindness in Experiment 2 when considering all conditions.

As we did for Experiment 1, we also ran a 2 (distractor type: positive, negative) × 2 (lag: 2, 4) × 2 (time manipulation condition: expansive, limited) mixed ANOVA. There was no impact of time manipulation when we isolated the analyses to these conditions: the distractor type × lag × time manipulation condition interaction, *F*(1,290) = 0.35, *p* = .556, $\eta_{p}^{2}=.001$, was non-significant. When the three-way interaction was isolated from the same distractor type × lag × time manipulation design using a Bayesian ANOVA, a Bayes factor of BF_10_ = 0.12 indicated moderate-to-strong evidence for the null hypothesis. In other words, the time manipulation had no effect on emotion-induced blindness in Experiment 2.

#### Questionnaire results

Table 2 represents questionnaire data from participants in Experiment 2.

On questionnaires, there was no difference between time manipulation condition groups on baseline mood, life progress, or future time perspective measures (*Fs* < 2.19, *ps* ≥ .113). However, across groups, there was a difference on the post-writing task mood, *F*(2,428) = 9.99, *p* < .001, $\eta_{p}^{2}=.05$, and mood change, *F*(2,428) = 5.59, *p* = .004, $\eta_{p}^{2}=.03$. Post-hoc *t*-tests revealed the expansive and limited conditions differed both on post-writing task mood, *t*(290) = 4.39, *p* < .001, *d* = .51, and mood change, *t*(290) = 3.07, *p* = .001, *d* = .36, as did the limited and control conditions (post-writing task mood, *t*(285) = 2.19, *p* = .029, *d* = .26; mood change, *t*(285) = 1.96, *p* = .049, *d* = .23). The expansive and control conditions also differed on the post-writing task mood, *t*(281) = 2.28, *p* = .023, *d* = .27, but not the mood change, *t*(281) = 1.45, *p* = .147, *d* = .17. These results indicate that those in the limited condition had significantly worse mood after the writing task compared to those in the expansive and control conditions, consistent with general patterns in Experiment 1 and with previous findings (Barber et al., 2016).

### Discussion

In both Experiments 1 and 2, the time perspective writing task made little difference on performance in the emotion-induced blindness task. This suggests that time perspective fails to penetrate at an attentional level, given that it did not replicate previous studies that have shown the effect in memory or in tasks that allow more time. Nevertheless, a possibility from the first two experiments is that our writing manipulation was ineffective. In this scenario, it may not be that the emotion blindness task is impenetrable to affective biases, but rather that our manipulation simply failed to produce affective biases via a shift in time perspective.

## Experiment 3

In Experiment 3, we incorporated a memory task. Based on previous literature and the results of our previous two experiments, we predicted that the time perspective manipulation would affect memory but not attention. We also chose to have all participants complete both the limited and expansive conditions. This allowed us to make within-subject comparisons to isolate the investigation of the effects of the writing task prompts as they impacted attention and memory within individuals.

### Method

#### Participants

297 individuals took part in Experiment 3, but data were excluded from seven because they did not complete the full experiment (*n* = 6) or completed a substantial number of trials before restarting the study to complete it fully (*n* = 1). An additional six datasets were excluded for individuals who did not report their age (*n* = 1) or who reported an age greater than 35 years old (*n* = 5). This resulted in 284 participants taking part in the study, identifying as 220 female, 58 male, 3 non-binary, 1 genderfluid, and 2 who preferred not to respond regarding their gender.^2^ Participants were aged 17–33 (*M* = 19.3, *SD* = 2.5) and were undergraduate students at the University of Western Australia, recruited through the School of Psychological Science Sona system between July and September 2024. Experiment 3 was not preregistered.

In Experiment 3, we aimed for a sample size of at least 254 participants. This was based on the main effect of time horizon ($\eta_{p}^{2}=.03$) in the memory results from Barber and Mather, Experiment 1 (Barber et al., 2016) using GPower (80% power, *α* = .05; Faul et al., 2007). We chose to oversample by at least 15%, expecting participants to be excluded for not completing the study or for poor performance on the tasks.

Experiment 3 took about 45 min to complete. It was approved by the University of Western Australia Human Ethics Board, protocol 2024/ET000413, “Emotion, Attention, and Perspective”. Participants were awarded course credit for their participation.

#### Materials

The materials and procedure used in Experiment 3 were similar to those used in Experiments 1 and 2, with the following exceptions. Importantly, participants completed two cognitive tasks: an emotion-induced blindness task and an emotional memory task. In Experiment 3, experimental tasks were programmed in Inquisit 6, 2019 and questionnaires and experiment instructions were presented in Qualtrics.

##### Time Perspective Writing Task.

To accommodate the within-subject design in Experiment 3, we reduced the time perspective conditions to only compare expansive and limited conditions, and no longer included a control time perspective condition. All participants completed the time perspective writing task twice: once answering the expansive condition questions before one half of the experiment, and once answering the limited condition questions before the other half of the experiment. Participants were explicitly told about the within-subject nature of the study. In the instructions for the study, participants were told: “In this experiment, you will answer some questions and complete the main tasks. Then, you will repeat by answering some different questions and completing the main tasks again. The questions will ask you to think about time in different ways. Please try to continue thinking about this as you complete the main tasks that follow them”. Participants were also instructed that there was open-ended response at the end of each condition, where they were told to “please write any additional thoughts/reactions that came to you about considering <preparing to live to 120 / having only 6 months left to live>”. This prompt was used to further encourage participants to continue to think about the time perspective (preparing to live to 120 or having only 6 months to live) throughout the tasks; responses to this prompt were not analysed.

##### Emotional Stimuli.

Additional images of negative and positive categories were included in Experiment 3 to accommodate the additional tasks. A set of 42 negative images (valence: *M =* 2.58, *SD =* .70; arousal: *M =* 6.25, *SD =* .59) and 42 positive images (valence: *M =* 7.28, *SD =* .56; arousal: *M =* 5.59, *SD =* .83) were of the same type as Experiments 1 and 2. Unlike the previous experiments, Experiment 3 did not include a “neutral” image condition. Images in Experiment 3 were not normalised for luminance using the SHINE Toolbox.

The sets of negative and positive images were split into six groups of seven images so that images could be counterbalanced across time perspective conditions and task types. Each participant saw each image in the experiment: 14 images on each emotion-induced blindness task (expansive and limited; 28 total) and 7 images on each emotional memory task (expansive and limited; 14 total). The images assigned to each task were counterbalanced between participants, so that specific images could appear in expansive or limited conditions and in either task type depending on the participants’ random assignment. Sets of images were made to have similar levels of valence (negative mean range: 2.51–2.73; positive mean range: 7.14–7.42) and arousal (negative mean range: 6.20–6.32; positive mean range: 7.14–7.42).

##### Emotional Memory Task.

The emotional memory task was based on closely on the design used by Barber et al. (2016). Seven negative and seven positive images were shown one at a time in a random order, with the restriction that no more than three of an image valence category would repeat in a row. Each trial began with a fixation cross for 1000 ms, followed by an image presented in the centre of the screen for 2000 ms, before repeating for the rest of the images. Participants were told to “Watch the images as if they were being shown on television. After you see all of the images, you will be asked to remember them by typing their descriptions”. After all images were shown, participants were presented with a blank textbox and instructed to write a brief description of each picture, such that “each description should be detailed enough that the researcher can tell which picture from the set you are describing (e.g. “people” or “faces” is not specific enough)”.

Participant responses on the emotional memory task were coded by B.L.K. Recalled descriptions that matched presented images were counted as “hits” and tallied for negative and positive images in expansive and limited conditions for each participant. When participants responded too vaguely to assign to a particular image, repeated a description of an image, falsely remembered an image to be present when it was not, or misremembered the details of the image that changed its implied valence (e.g. reported an image of a boy smiling when he was crying), we separately tallied these responses as “close matches”. This allowed us to additionally analyse results when close matches were considered; doing so revealed no change in the pattern of results.

##### Emotion-Induced Blindness Task.

Each emotion-induced blindness task included 42 trials. Unlike in Experiments 1 and 2, all trials in the emotion-induced blindness tasks were lag 2 trials, and emotional distractor conditions were negative, positive, or no distractor trials. Each emotion-induced blindness task was shown in two blocks of 24 trials, giving participants a break halfway through the trials.

##### Questionnaires.

In Experiment 3, participants completed the demographic, life progress, future time perspective, time orientation, and time organization questionnaires. Unlike previous experiments, they were not asked about their mood to limit the time of the experiment given the many conditions for each participant.

### Procedure

After consenting to the study, participants first completed the questionnaires. Then after reading the experiment instructions, participants completed the practice trials for the emotion-induced blindness task, so that they could get used to the complicated task before starting the study. Then, participants completed their first time perspective writing task. The order of the time perspective writing task conditions and cognitive task conditions was counterbalanced across participants, so that participants either completed the limited or expansive condition first or vice versa, and completed the emotion-induced blindness or emotional memory task first within each category or vice versa. After completing the first writing task, participants completed the emotion-induced blindness task and emotional memory task (in the order dependent on their counterbalanced condition). Participants then completed the final prompt from the previous writing condition before starting the next time perspective condition and the cognitive tasks in the same order as the first set of blocks. At the end of the study, participants were debriefed about the purposes of the study and assigned course credit for their participation.

### Results

#### Data screening

16 participants were excluded for poor overall performance accuracy on the emotion-induced blindness tasks (< 55%). An additional 10 participants were excluded for overall performance on the emotional memory task (no clear matches in either the limited and/or expansive condition). The final sample had 259 participants.

#### Writing task attention check

In both time manipulation writing tasks, all participants responded to prompts appropriately. In the expansive-future writing task, participants took an average of 210.2 s (*SD* = 154.5 s, range: 39.5–1072.5 s) and wrote 401.7 characters (*SD* = 252.7 characters, range: 47–1964 characters). In the limited-future writing task, participants took an average of 215.7 s (*SD* = 218.6 s, range: 20.5–2335.8 s) and wrote 411.5 characters (*SD* = 306.0 characters, range: 39–2754 characters).

#### Effect of time manipulation on emotional memory

**Figure 4.**
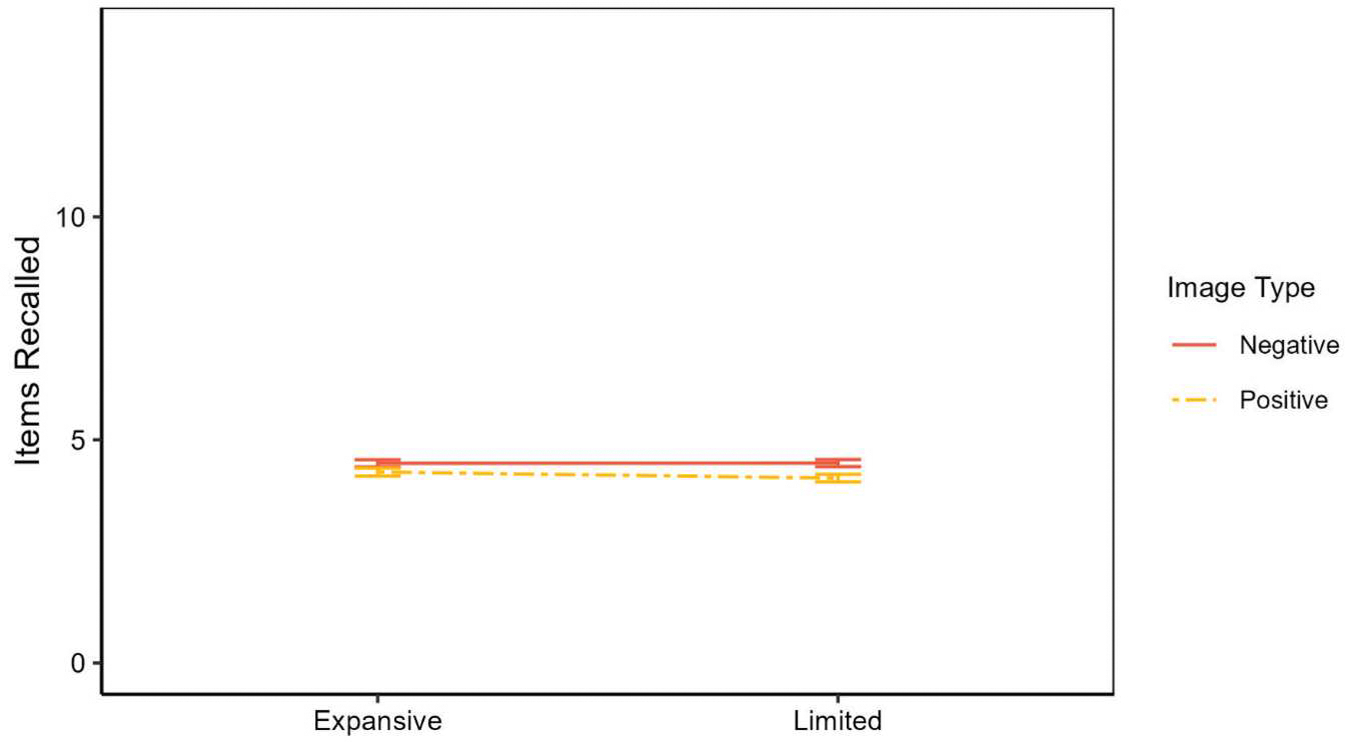
Experiment 3 emotional memory performance. Notes: Error bars represent within-subject standard error. For reference, means for each condition are also reported in the Supplemental Material Table S3.

We first examined how participants recalled images that were negative and positive in the different time manipulation conditions (Figure 4). A 2 (image type: positive, negative) × 2 (time manipulation condition: expansive, limited) within-subjects ANOVA revealed a significant main effect of image type, *F*(1,258) = 11.78, *p* < .001, $\eta_{p}^{2}=.04$, such that overall, participants remembered more negative than positive images. However, there was no significant main effect of time manipulation condition, *F*(1,258) < 1, *p* = .352, $\eta_{p}^{2}<.01$, nor image type × time manipulation condition interaction, *F*(1,258) < 1, *p* = .344, $\eta_{p}^{2}<.01$. These results indicated that the time manipulation did not affect emotional memory in our study. A Bayesian ANOVA confirmed no evidence for the image type × time manipulation condition interaction, BF_10_ = 0.17, indicating moderate evidence for the null hypothesis.

Given the within-subjects design of the study, we considered whether the order of the time perspective prompts or the order of tasks affected which images participants remembered. We thus examined whether completing a time perspective condition or task early in the experiment made a difference in the way time perspective influenced the results. We included these factors in a subsequent 2 (image type: positive, negative) × 2 (time manipulation condition: expansive, limited) × 2 (manipulation order: expansive first, limited first) × 2 (task order: emotion-induced blindness first, memory task first) ANOVA with number of images recalled as the dependent variable, and found significant interactions between time manipulation condition × manipulation order, *F*(1,255) = 8.41, *p* = .004, $\eta_{p}^{2}=.03$, and image type × time manipulation condition × manipulation order, *F*(1,255) = 11.88, *p* < .001, $\eta_{p}^{2}=.05$. In other words, participants’ performance was different if they did the expansive condition first versus limited condition first. There were no interactions with task order (*ps* ≥ .160).

**Figure 5.**
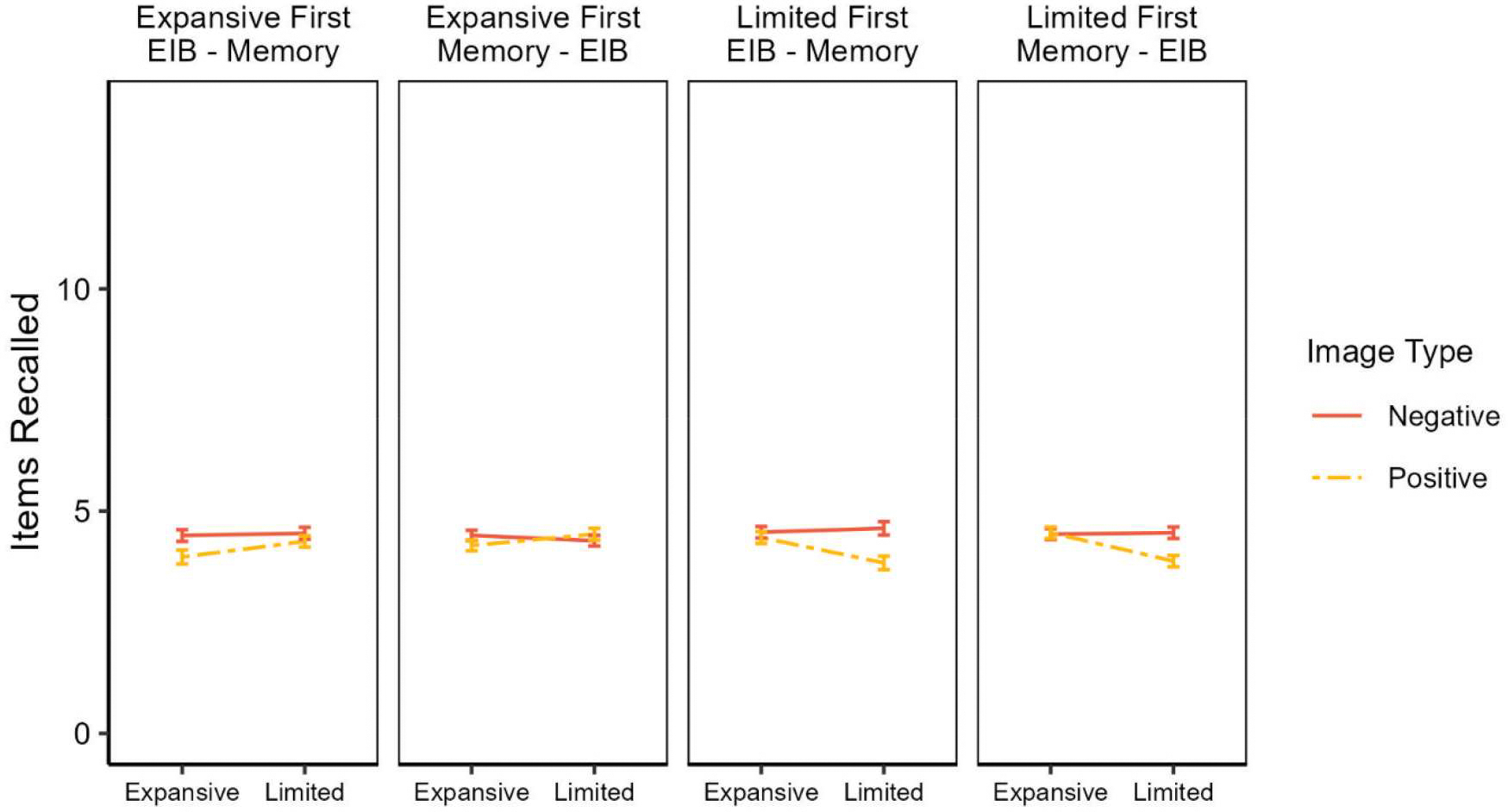
Experiment 3 emotional memory performance considering manipulation order and task order. Notes: Error bars represent within-subject standard error. “EIB-Memory” indicates performance for participants who completed the emotion-induced blindness task before the emotional memory task, and vice versa. “Expansive First” indicates performance from participants who completed the expansive condition before the limited condition and vice versa.

To account for these between-subjects differences, we ran subsequent post-hoc 2 × 2 ANOVAs for each of the manipulation and time order combinations with number of images recalled as the dependent variable (Figure 5). We found no image type × time manipulation condition interaction for participants who completed the expansive condition first (*ps* > .193); however, the 2 × 2 interaction was significant for participants who completed the limited condition first, regardless of whether they first completed the emotion-induced blindness task, *F*(1,57) = 4.53, *p* = .038, $\eta_{p}^{2}=.07$, or memory task, *F*(1,65) = 7.33, *p* = .012, $\eta_{p}^{2}=.09$. The pattern of these results was opposite to what we predicted: while we expected participants to remember relatively more positive images than negative images in the limited condition, participants who completed the limited condition first remembered fewer positive images than negative images in the limited condition (*ts* > 3.08, *ps* < .003), and showed no difference between negative and positive images in the expansive condition (*ts* < 1, *ps* > .576).

#### Effect of time manipulation on emotion-induced blindness

**Figure 6.**
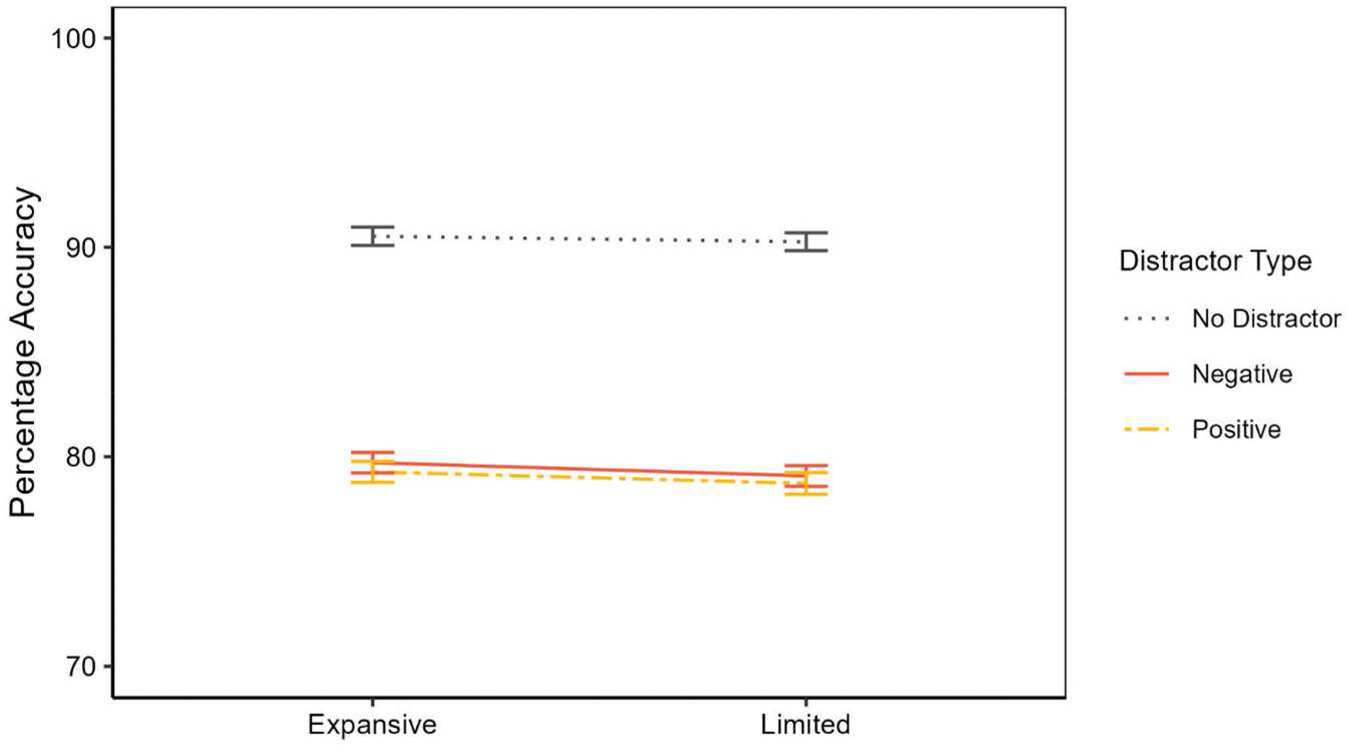
Experiment 3 emotion-induced blindness performance. Notes: Error bars represent within-subject standard error. For reference, means for each condition are also reported in the Supplemental Material Table S4.

We next analysed emotion-induced blindness performance (Figure 6). A 3 (distractor type: positive, negative, no distractor) × 2 (time manipulation condition: expansive, limited) mixed ANOVA revealed a significant main effect of distractor type, *F*(2,516) = 283.55, *p* < .001, $\eta_{p}^{2}=.52$, but no significant main effect of time manipulation condition, *F*(1,258) < 1, *p* = .348, $\eta_{p}^{2}<.01$, nor significant interaction between them, *F*(2,516) < 1, *p* = .907, $\eta_{p}^{2}<.01$. By reducing comparisons to be just between negative and positive distractors in a 2 (distractor type: negative, positive) × 2 (time manipulation condition: expansive, limited) ANOVA, there was no main effect of distractor type, time perspective, or interaction between them (*Fs* < 1, *ps* ≥ .347). The same 2 × 2 ANOVA using Bayesian methods revealed moderate-to-strong evidence for the null hypothesis in the distractor type × time manipulation interaction, BF_10_ = 0.11.

When we considered the role of between-subject manipulation order and task order in a 2 (distractor type: negative versus positive) × 2 (time manipulation condition) × 2 (manipulation order) × 2 (task order) ANOVA, there was a significant time perspective × manipulation order interaction, *F*(1,255) = 34.12, *p* < .001, $\eta_{p}^{2}=.12$, although no other interactions with these variables were significant (*ps* ≥ .097).

**Figure 7.**
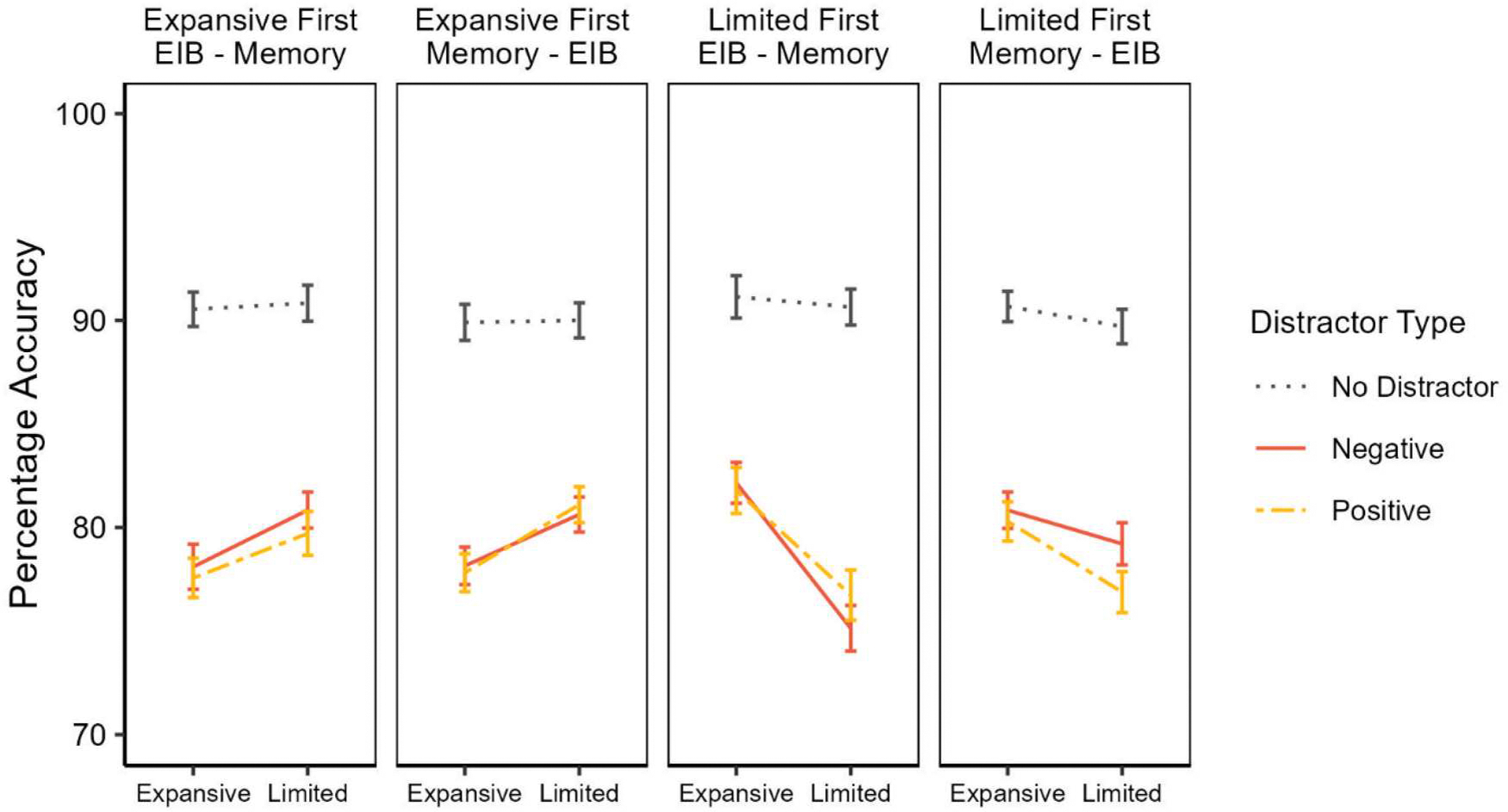
Experiment 3 emotion-induced blindness performance considering manipulation order and task order. Notes: Error bars represent within-subject standard error. “EIB-Memory” indicates performance from participants who completed the emotion-induced blindness task before the emotional memory task, and vice versa. “Expansive First” indicates performance from participants who completed the expansive condition before the limited condition and vice versa.

Breaking this down further into separable 2 × 2 comparisons for each manipulation order and task order combination (Figure 7), we found that the time perspective × manipulation order was not significant for any condition (*Fs* < 1, *ps* ≥ .352). Together, these data indicate that performance on the emotion-induced blindness task was worse after the expansive condition when the expansive condition came first, and worse after the limited condition when the limited condition came first; however, the relationship between negative and positive distractors was not different between expansive and limited conditions even when between-subject task ordering was considered.

## General discussion

Some theories about the positivity effect in older adults propose it to derive from a perspective of a limited time horizon (Carstensen, 2006; Reed & Carstensen, 2012). Consistent with this, previous research indicates that altering time perspective in younger adults can result in a similar priority for positive over negative emotion (Barber et al., 2016). Across three experiments, we examined the role of time perspective in younger adults as they completed an emotion-induced blindness task. We found minimal impact of manipulated time horizon perspectives across experiments. In all experiments, there was no significant impact of time perspective on emotion-induced blindness patterns. In Experiment 3, we included a recall memory task to determine if time perspective effects in memory would replicate previous research; however, we found a pattern opposite to expectations, such that younger adults were more likely to remember negative than positive images in a limited time perspective condition. Overall, our study contributes to the mixed literature about the role of time perspective in the positivity effect.

We used a time perspective manipulation across three direct replication manipulations with high power and did not find an impact on whether participants were more distracted by positive versus negative distractors in any experiment. Our main research question was whether time perspectives could influence attention when there was little availability for reflective processes to interfere with image processing, particularly because older adults do show the positivity effect within this type of fast attention task (Kennedy et al., 2020). The results for this study are congruent with the possibility that time perspectives may require more than 100 ms to influence performance. However, since we did not replicate previous positivity-shifts in memory (Barber et al., 2016), these results instead suggest that our time perspective manipulation failed to have the same impact as in prior work.

We used a within-subjects manipulation in Experiment 3 and found that the order of manipulations made a difference in the way negative versus positive images were remembered. When participants completed the expansive prompt first, they showed no impact of writing task manipulation in their recall for positive versus negative images, but when participants completed the limited prompt first, they recalled fewer positive images than negative images in the limited compared to expansive condition. In this order condition, the outcome is opposite to previous research (Barber et al., 2016), and it is not clear why. We consider some possibilities in the paragraphs below. While the order of manipulation also made a difference in attention results, we suspect that this effect was due to practice on the task. General performance on the emotion-induced blindness task was better in the second manipulation condition than in the first, indicating that performance likely benefited from experience on the task rather than an effect of the manipulations themselves. Similar to previous studies, mood was somewhat affected by the writing task manipulations in Experiment 1 and 2, such that those in the limited time perspective condition had patterns of worse mood compared to the other conditions.

There are several reasons why this manipulation may not have had effects on memory recall in our study in the way it has in previous research. One possibility is that the task simply did not manipulate time horizons as intended. Although the time manipulation exercise has been successful at inducing the positivity effect in younger adults (Barber et al., 2016), flaws in the design have been noted before. For example, Barber and Kim suggest that the exercise elicits more meaningful reflection and words related to emotional goals, such that it may not be the time horizon per se but salience to such meaningful concepts that creates a positivity effect (Barber & Kim, 2022). We also found that several participants answered our prompts in ways that were thoughtful and appropriate to the prompts but in a manner differently than expected. For example, several participants in the “expansive” condition indicated they would spend the lengthy amount of time left in their life with their loved ones. Since this is a response more congruent with the expectations of a “limited” time perspective, such an array of responses to the prompts may have muddled the influence of the time manipulation writing task across conditions. Younger adults may also interpret the idea of dying in six months in a substantially different way from older adults, given differences in experience and the time they have already lived. For example, although in Experiment 3 we emphasised to participants to continue to think about the writing prompts while completing the tasks, the potency of the manipulation seemed most evident at the beginning of the experiment; these young adult participants may have thought about dying in six months in a way dominated by negative feelings. Differences that we found in the ordering of the tasks may indicate that participants failed to fully process those thoughts before starting the tasks, or that the impacts of the manipulation subsided as the experiment progressed.

It may also be the case that we observed opposite effects from Barber et al. (2016) because we directed participants’ attention to the relationship between the time perspective exercise and the subsequent tasks, whereas in Barber et al., these were presented as independent tasks. The most intuitive assumption is that focusing on the possibility of dying soon will lead to negative mood and so negative biases in attention. Our participants may have been influenced by this conscious interpretation of the writing exercise, whereas if they had not thought about it explicitly, the limited time perspective exercise might have been more effective in leading them to attempt to regulate emotion and optimise their more limited time.

Another possibility for a lack of effects from the time manipulation is that the manipulation may not be capable of affecting performance with more demanding tasks. Our experiment was more complicated than previous designs and likely required more cognitive resources from participants, which may have diluted or changed the effect of the manipulation when participants became preoccupied with the task. Notably, our results contrast with other findings that show effects of time perspective in attention (Pruzan & Isaacowitz, 2006; Zsoldos & Hot, 2024). For example, Zsoldos and Hot (2024) recently found that older adults and young adults in a limited time perspective biased toward positive over negative stimuli, but young adults in the control condition did not. However, they allowed more time between the emotional stimuli and task-relevant probes, which may have permitted additional influence from the time perspective manipulation. It is worth noting that even the findings from Zsoldos and Hot (2024) showed only modest differences in performance between young adults in a limited time perspective condition compared to young adults in a control condition, with both conditions showing a similar pattern of positive bias compared to negative bias.

Another important consideration is that the experiments in this study were run online. The online design choice provided many strengths, such as allowing us to power our studies appropriately and collect data from many participants in a concentrated timeframe. Nevertheless, this meant that the experimental control of some aspects in our study was lower than in the lab; for example, we did not have control over the computers or monitors that participants used to complete the task, nor did we have control over the environment where they completed the experiment. We have used similar online studies to observe positivity effects in emotion-induced blindness that closely mimic patterns we observe in the lab (e.g. Kennedy et al., 2020) and previous research has shown effects of the time perspective manipulation using similar online samples (e.g. Barber et al., 2016). The task-relevant responses to the time manipulation prompts (although sometimes not as expected), as well as the high percentage of accuracies that mimic similar emotion-induced blindness studies, indicate that participants were on task when they completed this experiment. However, we note that experimental conditions, as well as problems with the manipulation itself, may have contributed to the generally null effects of the time perspective writing manipulation. Future research may do well to determine the boundary conditions of the time manipulation and the constructs it is capable of assessing.

Overall, the positivity effect represents a window into mechanisms that tend to change in older adulthood that could provide keys to benefit individuals that struggle with negative emotions. Understanding the underlying mechanisms involved is thus an important pursuit, and time perspective – in some form – may very well be involved in such patterns. Our results add to the complicated literature related to time perspective and the positivity effect. Across three studies, we found that time perspective manipulations did not impact younger adults’ attention to emotional stimuli in an emotion-induced blindness task. We think this null effect is informative, as it does not provide strong support that age differences in time perspective drive the previously identified age-by-valence interaction in emotion-induced blindness. Future research should consider other factors to account for such effects (for one such alternative possibility, see Mather, 2024), and these results invite more work to clarify when and how time perspective will influence affective biases.

## Supplementary Material

Supp

Supplemental data for this article can be accessed online at https://doi.org/10.1080/02699931.2026.2675414.

## Figures and Tables

**Table 1.** Means and 95% Confidence Intervals of questionnaire data for each time perspective condition in Experiment 1

	Expansive condition	Limited condition	Control condition

Baseline mood	55.54 [51.14, 59.93]	57.22 [52.03, 62.41]	57.00 [52.33, 61.67]
Post-writing task mood	59.76 [55.36, 64.15]	53.78 [49.00, 58.57]	58.14 [53.91, 62.38]
Mood change*	4.22 [1.38, 7.06]	−3.44 [−7.77, 0.90]	1.14 [−1.53, 3.82]
Life progress	22.55 [19.18, 25.92]	24.12 [21.10, 27.14]	23.72 [20.83, 26.61]
Future perception	4.78 [4.49, 5.06]	4.82 [4.56, 5.07]	5.08 [4.88, 5.29]

* Indicates significance at the *p* < .05 level.

**Table 2.** Means and 95% Confidence Intervals of questionnaire data for each time perspective condition in Experiment 2.

	Expansive condition	Limited condition	Control condition

Baseline mood	62.06 [58.64, 65.47]	58.91 [55.35, 62.47]	59.91 [56.54, 63.27]
Post-writing task mood*	66.14 [63.29, 68.99]	56.45 [53.16, 59.75]	61.39 [58.41, 64.37]
Mood change*	4.08 [1.38, 6.78]	−2.46 [−5.67, − 0.75]	1.48 [−0.79, 3.76]
Life progress	22.39 [20.64, 24.13]	24.20 [22.13, 26.28]	25.30 [23.28, 27.33]
Future perception	5.01 [4.84, 5.19]	4.88 [4.71, 5.06]	4.89 [4.71, 5.08]

* Indicates significance at the *p* < .05 level.

## Data Availability

The anonymized data and experimental programmes presented in this study are openly available and can be downloaded at: https://osf.io/da84u.

## Footnotes

1 We preregistered our Experiment 2 based on findings of Experiment 1. However, in the preregistration, we used data without eliminating one participant who did not complete the time perspective writing task manipulation (see Data Screening). The results with this participant included initially indicated a small but significant 2 (positive, negative) × 2 (expansive, limited) interaction in the emotion-induced blindness data. However, when that participant was removed for the final analyses (as they did not complete the requested task), that interaction no longer revealed a significant effect. The effect size to determine the participant number in Experiment 2 changed a trivial amount, however the perspective with which we composed our preregistration was to further investigate a significant difference that was no longer significant with this participant removed.

2 Although gender representation was imbalanced within each experiment, there were no significant interactions between gender and time manipulation condition in any of the experiments.
