# Supplementary material for: About time? The role of time perspective in the priority for positive over negative emotion in attention
Source: Cogn Emot. Author manuscript; Available in PMC 2026 Aug 16. (PMC13477811; doi:10.1080/02699931.2026.2675414)
Supplement: Supplementary file 1 [file NIHMS2197335-supplement-Supp.docx]

Supplemental Material:

About time? The role of time perspective in the priority for positive over negative emotion in attention

Briana L. Kennedy^1,*^ & Mara Mather^2^

^1^ School of Psychological Science, University of Western Australia

^2^ Leonard Davis School of Gerontology, Department of Psychology, and Department of Biomedical Engineering, University of Southern California

**Performance Descriptive Statistic Tables**

Figures in our manuscript depict the averaged performance for time manipulation groups and semester groups. For reference, we include tables here with the same data. Supplemental Table S1 indicates the means and 95% confidence intervals in emotion-induced blindness performance for each group condition analyzed in Experiment 1.

**Table S1**
*Mean percentage accuracy and 95% confidence intervals of emotion-induced blindness performance for each group condition in Experiment 1*

| ***Negative, Lag 2*** | | | | ***Negative, Lag 4*** | | | |
| --- | --- | --- | --- | --- | --- | --- | --- |
|  | Expansive | 75.46 | [72.99, 77.93] |  | Expansive | 83.64 | [80.87, 86.40] |
|  | Limited | 77.24 | [74.13, 80.36] |  | Limited | 83.07 | [80.20, 85.94] |
|  | Control | 78.62 | [76.38, 80.85] |  | Control | 86.73 | [84.37, 89.09] |
| ***Neutral, Lag 2*** | | | | ***Neutral, Lag 4*** | | | |
|  | Expansive | 84.15 | [81.39, 86.90] |  | Expansive | 88.67 | [86.46, 90.88] |
|  | Limited | 84.67 | [82.05, 87.29] |  | Limited | 87.66 | [85.05, 90.27] |
|  | Control | 86.95 | [84.68, 89.22] |  | Control | 90.79 | [88.71, 92.87] |
| ***Positive, Lag 2*** | | | | ***Positive, Lag 4*** | | | |
|  | Expansive | 75.46 | [72.87, 78.05] |  | Expansive | 82.67 | [79.43, 85.91] |
|  | Limited | 74.63 | [71.84, 77.41] |  | Limited | 84.46 | [81.82, 87.09] |
|  | Control | 75.55 | [72.59, 78.50] |  | Control | 87.28 | [84.96, 89.60] |
| ***No Distractor, Lag 2*** | | | | ***No Distractor, Lag 4*** | | | |
|  | Expansive | 87.70 | [85.14, 90.26] |  | Expansive | 89.43 | [86.85, 92.01] |
|  | Limited | 86.43 | [83.52, 89.35] |  | Limited | 88.14 | [85.73, 90.55] |
|  | Control | 91.34 | [89.34, 93.33] |  | Control | 91.94 | [89.88, 94.01] |

Similarly, Supplemental Table S2 indicates the means and 95% confidence intervals in emotion-induced blindness performance for each group condition analyzed in Experiment 2.

**Table S2**
*Mean percentage accuracy and 95% confidence intervals of emotion-induced blindness performance for each group condition in Experiment 2*

| ***Negative, Lag 2*** | | | | ***Negative, Lag 4*** | | | |
| --- | --- | --- | --- | --- | --- | --- | --- |
|  | Expansive | 77.72 | [75.64, 79.80] |  | Expansive | 86.37 | [84.66, 88.09] |
|  | Limited | 76.60 | [74.73, 78.48] |  | Limited | 85.92 | [84.15, 87.70] |
|  | Control | 75.60 | [73.71, 77.49] |  | Control | 84.68 | [82.77, 86.60] |
| ***Neutral, Lag 2*** | | | | ***Neutral, Lag 4*** | | | |
|  | Expansive | 87.15 | [85.46, 88.84] |  | Expansive | 90.22 | [88.80, 91.64] |
|  | Limited | 86.43 | [84.73, 88.13] |  | Limited | 89.81 | [88.40, 91.21] |
|  | Control | 84.20 | [82.05, 86.35] |  | Control | 89.15 | [87.35, 90.95] |
| ***Positive, Lag 2*** | | | | ***Positive, Lag 4*** | | | |
|  | Expansive | 76.68 | [74.89, 78.47] |  | Expansive | 86.55 | [84.93, 88.16] |
|  | Limited | 77.14 | [75.34, 78.94] |  | Limited | 86.60 | [84.97, 88.23] |
|  | Control | 76.29 | [74.17, 78.41] |  | Control | 84.53 | [82.65, 86.42] |
| ***No Distractor, Lag 2*** | | | | ***No Distractor, Lag 4*** | | | |
|  | Expansive | 90.83 | [89.31, 92.35] |  | Expansive | 91.29 | [89.84, 92.74] |
|  | Limited | 90.91 | [89.49, 92.32] |  | Limited | 90.12 | [88.69, 91.55] |
|  | Control | 88.40 | [86.60, 90.20] |  | Control | 90.14 | [88.48, 91.80] |

Finally, memory performance and emotion-induced blindness performance from Experiment 3 are displayed in Tables S3 and S4 respectively.

**Table S3**

*Mean percentage accuracy and 95% confidence intervals of memory performance for each condition in Experiment 3*

| ***Negative Recall*** | | | |
| --- | --- | --- | --- |
| Expansive | 4.48 | [4.32, 4.63] |  |
| Limited | 4.48 | [4.32, 4.63] |  |
| ***Positive Recall*** | | | |
| Expansive | 4.28 | [4.10, 4.46] |  |
| Limited | 4.14 | [3.97, 4.31] |  |

**Table S4**

*Mean percentage accuracy and 95% confidence intervals of emotion-induced blindness performance for each condition in Experiment 3*

| ***Negative, Lag 2*** | | | |
| --- | --- | --- | --- |
| Expansive | 79.72 | [78.29, 81.15] |  |
| Limited | 79.08 | [77.64, 80.52] |  |
| ***Positive, Lag 2*** | | | |
| Expansive | 79.27 | [77.83, 80.72] |  |
| Limited | 78.72 | [77.25, 80.20] |  |
| ***No Distractor, Lag 2*** | | | |
| Expansive | 90.53 | [89.34, 91.71] |  |
| Limited | 90.26 | [88.95, 91.58] |  |

**Individual Differences**

In addition to personal time horizons, other individual differences can impact positivity effect patterns. Some studies have examined individual differences in time perspective related to emotional processing, with inconsistent results (Barber & Kim, 2022; Gronchi et al., 2018). Nevertheless, some previous research indicates that individual differences in self-reported time horizons (i.e., if someone estimates they have less, rather than more, left in life; e.g., Carstensen & Reynolds, 2023; Shavit et al., 2023, see also Barber & Kim, 2021) and time orientations (i.e., if someone has a tendency to view the past, present, or future in a more “balanced” way; Ceccato et al., 2021) can correlate with positivity effect patterns. Several studies indicate that mood can also predict the positivity effect in a way complimentary to time horizons (Barber et al., 2016; Isaacowitz et al., 2008), and others indicate that negative emotional tendencies or personality factors can correlate with time perspective (Grühn et al., 2016; Przepiorka et al., 2019). Some individual differences may also interfere with positivity effect patterns. For example, there remain possibilities that depression can reduce estimates of time left in life while also leading to negative biases in processing emotional stimuli, which could obscure any positive-bias effects of limited time perspective on emotional processing (Przepiorka et al., 2019). Altogether, time horizons and several individual differences seem to be possible mechanisms related to the effect, although their unique influence and interactions remain unclear (see Barber & Kim, 2021).

**Experiment 1**

Since there were no predicted group differences between time manipulation conditions, we chose to examine individual difference data outside of the assigned writing task groups in a post-hoc analysis. We started by examining if individual differences on the questionnaires correlated with the positivity effect, looking at all individuals regardless of their group assignment. To operationalize the positivity effect, we subtracted each individual’s positive lag 2 performance from their negative lag 2 performance to calculate their “positive-over-negative bias,” regardless of the time manipulation group that they were in. This gave us a score to represent how much each participant prioritized positive over negative distractors: the more positive a participant’s score, the more their data represented a pattern consistent with the “positivity effect.” Even without corrections for multiple comparisons, Pearson correlations revealed that there was no correlation between mood change and the positive-over-negative bias, *r* = .02, *p* = .758, nor a correlation between the positive-over-negative bias and life progress or future time perspective (*rs* ≤ .08, *ps* ≥ .213).

**Experiment 2**

In Experiment 2, we considered other individual differences related to affect and time. As we describe in more detail below, for affect, we included measures of positive and negative affect, dimensions of personality, and optimism/pessimism. Each measured unique qualities of affect that we predicted could relate to the way individuals prioritize positive versus negative stimuli. For constructs related to time, we included two additional measures. The measure that we used for time orientation was borrowed from an established questionnaire (Behaviorally Anchored Response Scales of Time Urgency; Landy et al., 1991) and relates to the way people use time in the way they approach daily activities. While not unrelated to the constructs measured in the time orientation questionnaire, we also developed a novel time organization questionnaire to capture the way people behave and organize their time, which we suspected may better capture the way we suspected people may prioritize time in the way they behave, and therefore the way they may approach the positivity effect task. The constructs that we measured in the time orientation and time organization questionnaires were different from time perspective as we manipulated in this experiment, which relates more specifically to how people view the amount of time left in their future.

Previous research motivated our choices. Individual differences in trait tendencies toward emotions have shown to correlate with the positivity effect (Grühn et al., 2016; Przepiorka et al., 2019), motivating a measure of affect, above and beyond mood, to be included in Experiment 2. Additionally, recent research indicates that individual differences in time orientation in younger adults (i.e., having a “balanced time perspective”) protect them from pessimistic views for the future (Ceccato et al., 2021). In other words, one’s relationship with time may also be a driver in the way that one biases toward emotions. This would be consistent with the idea that individuals who chose to participate in Experiment 1 in the beginning of the semester had a different, more balanced, orientation toward time compared to other participants, making them more likely to show positivity biased patterns. Altogether, in line with these previous individual difference findings, and based on the semester effects in Experiment 1, we included additional questionnaires in Experiment 2 to capture these constructs. We approached analyses of individual differences with hypotheses that participants with high trait time orientation, low pessimism, and high positive affect would exhibit patterns more consistent with a positivity bias than those on the other sides of such spectrums.

Below we describe the additional questionnaires used in Experiment 2. For each, we report Cronbach α values for reliability, which were calculated using the *Psych* package in *R.*

The Positive and Negative Affect Schedule (PANAS; Watson & Clark, 1994) was used as a measure of negative and positive affect. The 20-item scale included a list of ten negative words and ten positive words. Participants were asked to indicate the extent to which they felt over the past week on a scale from 1 (very slightly or not at all) to 5 (extremely). The 20-item questionnaire demonstrated good reliability, with Chronbach’s α = .71.

The Big Five Inventory (BFI; John et al., 1991, 2008) served as a measure of different dimensions of personality. The questionnaire included 44 statements to represent constructs of openness, conscientiousness, extroversion, agreeableness, and neuroticism. For each item, participants responded to the prompt “I am someone who…” on a scale from 1 (disagree strongly) to 5 (agree strongly). Using all 44 items, internal reliability was good, with Cronbach’s α = .72.

The Revised Life Orientation Test (LOT-R; Scheier et al., 1994) was used as a measure of optimism/pessimism. The 10-item questionnaire included six items related to optimism/pessimism, and another four questions that serve as fillers to make the purpose of the questionnaire less obvious. Participants rated each item on a scale from 0 = strongly disagree to 4 = strongly agree. Reliability of the 10-item questionnaire was good, with Cronbach’s α = .75.

As a measure of time orientation, a subset of questions from the Behaviorally Anchored Response Scales of Time Urgency (Landy et al., 1991) were used to determine the extent that individuals engage with time as they plan their daily activities. We chose seven questions from this questionnaire related to awareness of time, scheduling, list making, deadline control, speech patterns, nervous energy, and eating behavior. Participants made their response on a scale from one to seven. Anchors for the scale were phrased differently for each question but generally ranged from values from a low awareness to high awareness of time. Notably, the reliability of the time urgency scale was poor (Cronbach’s α = .51). This could be because of the subset of questions that we chose from the questionnaire, or due to the broad nature of questions that the questionnaire captured.

As a measure of time organization, we included seven novel questions about the way individuals describe their behaviour in relation to time (e.g., how often they arrive early, procrastinate, look at a clock). Participants rated how much time-related activities described their behavior on a scale from 1 (disagree strongly) to 5 (agree strongly). The time organization questionnaire is available in our supplementary materials available on OSF: https://osf.io/da84u. The time organization questionnaire demonstrated questionable reliability, although still higher than the time orientation questionnaire, with Cronbach’s α = .63.

We examined how these different individual difference measures correlated with how much participants were impacted by positive compared to negative stimuli. We again used an approach to calculate a positive-over-negative bias by subtracting positive lag 2 trial performance from negative lag 2 trial performance and examined the relationship across all participants regardless of their time manipulation group or semester that they completed the study (since time perspective manipulations made no difference to performance). Even with no corrections applied for multiple comparisons, Pearson correlations revealed no relationship with the PANAS (positive or negative), any of the Big Five factors (extroversion, agreeableness, conscientiousness, neuroticism, openness), optimism/pessimism, baseline mood, post-writing task mood, mood change, life progress score, or future time perspective. There were, however, two individual difference measures that did correlate with the positive-over-negative bias (at least without corrections for multiple comparisons), and both were our time-related questionnaires. Time orientation, *r* = .11, *p* = .030, and time organization, *r* = .10, *p* = .039, were two measures that positively correlated with the positive-over-negative bias. Note that with corrections for multiple comparisons, neither relationship was significant. However, both were in a direction consistent with our prediction based on Experiment 1, such that those with more concern for time had more of a bias for positive-over-negative. Overall, we found some evidence in Experiment 2 for relationships between participants’ bias toward positive over negative information and individual differences on two time-related questionnaires, such that those with more concern for time were more affected by positive compared to negative distractors.

**Experiment 3**

In Experiment 3, we calculated individual positive-over-negative bias for memory and emotion-induced blindness performance overall by collapsing across expansive and limited conditions for each task. Pearson correlations revealed no relationships with life progress, time orientation, time organization, or future time perspective in either memory or attention, even before correcting for multiple comparisons.

**Individual Differences Summary**

We found minimal effects of individual differences, but future research could further expand investigation into individual differences that relate to the positivity effect in attention. For example, previous research in the positivity effect research has found relationships between the effect and depression (e.g., Przepiorka et al., 2019). Past research has showed mixed evidence for the relationship between emotion-induced blindness and depression and anxiety (see Goodhew & Edwards, 2022), and our previous study with younger and older adults indicated no relationship between these constructs and the positivity effect in emotion-induced blindness (Kennedy et al., 2020). However, future research may particularly explore individual differences related to both affect and time perspective for a richer understanding of what makes some individuals more prone on this task to be affected by certain images. We also chose to only examine younger adults in this study to examine the effect of time horizons outside of age, however future studies may increase the scope of investigation to determine how time manipulations affect attention to emotional stimuli in both younger and older adults.

**Time of Semester**

**Experiment 1**

In a post-hoc analysis of the data, we considered a time horizon other than time left in life. Previous research indicates that undergraduate students in their senior year of college paid attention to negative information less and reported positive affect more than students in their first year (Pruzan & Isaacowitz, 2006). In a similar vein, we wondered if the semester may be an experience of time with an expansive start and approaching end. We therefore chose to – rather than by writing task manipulation – instead divide participants based on when they chose to participate in our study rather than by time manipulation groups: with the middle of the semester designated as March 18, 2018 (which marked the first day back after spring break), 106 participants took part in the experiment in the first half of the semester, and 130 took part in the second half of the semester (see Figure S1). These groups did not differ in their number of years of education, in their ratings of life progress, or in their ratings of future perception, *ts* ≤ 1.46, *ps* ≥ .145.

**Figure S1**

*Experiment 1 exploratory emotion-induced blindness results from time of semester*

*Note:* Error bars represent between-subject standard error. For reference, means for each condition are also reported in the Supplemental Material Table S5.

A 4 (distractor type: positive, negative, neutral, no distractor) × 2 (lag: 2, 4) × 2 (semester time: first half, second half) mixed ANOVA revealed no main effect of semester time, *F*(1,234) = 0.04, *p* = .835, *η^2^_p_* < .001. However, there was a significant interaction between distractor type and semester time, *F*(3,702) = 2.81, *p* = .039, *η^2^_p_* = .01. There was no significant interaction between lag and semester time, *F*(1,234) = 0.63, *p* = .428 *η^2^_p_* = .003, but there was a three-way interaction between distractor type, lag, and semester time, *F*(3,702) = 2.94, *p* = .032, *η^2^_p_* = .01. Those that chose to complete the experiment earlier in the semester showed greater effect from positive distractors than negative distractors compared to those that chose to complete the experiment later in the semester. Further post-hoc *t*-tests at lag 2 indicated those that completed the experiment in the first half of the semester were more distracted by positive distractors than negative distractors, *t*(105) = 3.73, *p* < .001, *d* = .36. There was no difference at lag 4 in those that completed the experiment in the first half of the semester, *t*(105) = 0.43, *p* = .669, *d* = .04. Participants that completed the experiment in the second half of the semester showed no difference between the negative and positive distractor trials at either lag 2 or lag 4 (*ts* ≤ 0.92, *ps* ≥ .179).

**Table S5**

*Experiment 1 Semester Means and 95% Confidence Intervals*

| ***Negative, Lag 2*** | | | | ***Negative, Lag 4*** | | | |
| --- | --- | --- | --- | --- | --- | --- | --- |
|  | Semester 1 | 78.73 | [76.65, 80.82] |  | Semester 1 | 84.12 | [81.76, 86.46] |
|  | Semester 2 | 75.71 | [73.56, 77.85] |  | Semester 2 | 84.71 | [82.64, 86.78] |
| ***Neutral, Lag 2*** | | | | ***Neutral, Lag 4*** | | | |
|  | Semester 1 | 84.39 | [82.04, 86.75] |  | Semester 1 | 88.68 | [86.74, 90.62] |
|  | Semester 2 | 85.90 | [84.02, 87.78] |  | Semester 2 | 89.29 | [87.46, 91.13] |
| ***Positive, Lag 2*** | | | | ***Positive, Lag 4*** | | | |
|  | Semester 1 | 74.69 | [72.51, 76.86] |  | Semester 1 | 83.69 | [81.19, 86.18] |
|  | Semester 2 | 75.64 | [73.37, 77.91] |  | Semester 2 | 85.61 | [83.51, 87.71] |
| ***No Distractor, Lag 2*** | | | | ***No Distractor, Lag 4*** | | | |
|  | Semester 1 | 88.32 | [88.25, 85.98] |  | Semester 1 | 90.33 | [88.24, 92.42] |
|  | Semester 2 | 88.62 | [86.69, 90.56] |  | Semester 2 | 89.39 | [87.57, 91.21] |

**Experiment 2**

In Experiment 1, post-hoc analyses suggested that time of semester that participants chose to participate influenced the results, such that those that completed the experiment in the beginning of the semester performed with a greater positivity bias than those later in the semester. This pattern was unexpected, as we expected the opposite pattern when we approached this analysis. While speculative, this may reflect the way people allocate their time. Those that completed the task early in the semester may tend to do things in the present (with a “limited” trait time perspective, similar to older adults), whereas those that complete it later in the semester may approach the semester requirements with an “expansive” trait time perspective and complete their research only when the deadline was nearer. These results may be indicative of individual differences – particularly related to time use – that influence a positivity bias in younger adults. In Experiment 2, we sought to replicate the semester effects and predicted that like our previous study, those that completed the study early in the semester would show a positivity effect pattern compared to those that completed it late in the semester.

We examined if time of semester impacted the positivity effect in Experiment 2. Regardless of writing task condition, we separated participants by those who completed the experiment between October and the end of December in the second half of the fall semester, and those that completed the experiment in January to February in the first half of the winter semester. 182 participants took part in the study in the first half of their semester, whereas 249 participants took part in the study in the second half of their semester. These groups did not differ in their number of years of education, in their ratings of life progress, or in their ratings of future perception, *ts* ≤ 1.16, *ps* ≥ .124.

**Figure S2.** *Experiment 2 emotion-induced blindness results from time of semester*

*Note:* Error bars represent between-subject standard error. For reference, means for each condition are also reported in the Supplemental Material Table S6.

A 4 (distractor type: positive, negative, neutral, no distractor) × 2 (lag: 2, 4) × 2 (semester time: first half, second half) mixed ANOVA revealed no main effect of semester time, *F*(1,429) = 0.35, *p* = .557, *η^2^_p_* = .001, no interaction between distractor type and time of semester, *F*(3,1287) = 1.60, *p* = .187, *η^2^_p_* = .004, and no interaction between lag and time of semester, *F*(1,429) = 3.10, *p* = .079, *η^2^_p_* = .007 (see Figure S2). Notably, there was a significant distractor type × lag × semester time interaction, *F*(3,1287) = 3.06, *p* = .027, *η^2^_p_* = .007, such that those that completed the task in the first half of the semester, compared to the second half of the semester, were more impacted by positive distractors at the early lag but the opposite was true for the later lag. However, post-hoc tests revealed there was no difference between negative and positive conditions in either lag in either semester conditions, *ts* < 1.02, *ps* > .312. In other words, the semester effects that we found in Experiment 1 replicated in Experiment 2, although to a lesser extent.

**Table S6**

| ***Negative, Lag 2*** | | | | ***Negative, Lag 4*** | | | |
| --- | --- | --- | --- | --- | --- | --- | --- |
|  | Semester 1 | 76.15 | [74.70, 77.61] |  | Semester 1 | 86.40 | [85.11, 87.68] |
|  | Semester 2 | 77.34 | [75.58, 79.09] |  | Semester 2 | 84.68 | [82.98, 86.39] |
| ***Neutral, Lag 2*** | | | | ***Neutral, Lag 4*** | | | |
|  | Semester 1 | 85.09 | [83.69, 86.49] |  | Semester 1 | 89.49 | [88.27, 90.71] |
|  | Semester 2 | 87.13 | [85.49, 88.78] |  | Semester 2 | 90.06 | [88.78, 91.35] |
| ***Positive, Lag 2*** | | | | ***Positive, Lag 4*** | | | |
|  | Semester 1 | 76.84 | [75.41, 78.28] |  | Semester 1 | 85.63 | [84.28, 86.97] |
|  | Semester 2 | 76.53 | [74.84, 78.23] |  | Semester 2 | 86.31 | [84.88, 87.74] |
| ***No Distractor, Lag 2*** | | | | ***No Distractor, Lag 4*** | | | |
|  | Semester 1 | 89.79 | [88.55, 91.03] |  | Semester 1 | 90.28 | [89.09, 91.46] |
|  | Semester 2 | 90.45 | [89.11, 91.79] |  | Semester 2 | 90.84 | [89.57, 92.11] |

In both Experiment 1 and 2, we found semester effects that contradicted what we assumed previous research would predict. It was individuals in the first half of the semester – not second half of semester – that showed more of a priority for positive over negative stimuli. In other words, rather than the time horizon itself being limited, it was individuals that signed up for the study in the beginning of the semester that were less affected by negative and more affected by positive emotions. We suggest that this may indicate an individual difference in the participants that sign up for studies earlier rather than later in the semester. When we explored possible individual differences in Experiment 2 (see above), it was not mood change, personality factors, or positive or negative affect that predicted the positivity effect. Instead, it was individuals with more awareness and concern for time that showed a positivity effect. One’s relationship with time as an individual difference therefore seems to be the quality that our results cumulatively suggest responsible for positivity effect patterns in emotion-induced blindness. This is consistent with other time effects that have been found related to the positivity effect: for example, older adults tend to remember positive information as being presented more recently than negative information, indicating that older adults bias the closeness in time that positive events happen compared to negative events (Ceccato et al., 2022).

If semester effects were due to awareness and concern for time, then participants earlier in the semester should have higher levels of time orientation and time organization. However, we found that this was not the case. There was no difference in time orientation scores in participants that completed the experiment in the first half or second half of the semester, *t*(429) = 1.47, *p* = .143, *d* = .14. For time organization, there was a significant difference in participants that completed the experiment in the first half versus second half of the semester, *t*(429) = 3.54, *p* < .001, *d* = .35. However, this was the opposite of what we expected, such that participants that completed the experiment in the first half of the semester (*M* = 21.86, *SD* = 4.74) had *less concern* about time than those that completed the experiment in the second half of the semester (*M* = 23.42, *SD* = 4.86).

Since the underlying mechanism related to time of semester effects remain unknown and were not related to time orientation or time organization, we chose to leave these exploratory questions out of Experiment 3 to focus on time manipulations, which were the main goal of our research. Future research focused on time of semester or individual differences related to time may benefit by incorporating measures of both to see how they relate or do not relate to one another, and in experimental designs better targeted to understand their possible mechanisms. Future research may also incorporate additional candidate individual differences including more clinically related constructs, such as depression, anxiety, or attention deficit hyperactivity disorder (ADHD), which may also predict behavior related to time use. As it stands, our results indicate that the factors of time that we measured in this experiment and the positivity effect may be related but are not conclusive.
